# Editorial: Proceedings of the 4th biennial conference of the International Society for Plant Molecular Farming

**DOI:** 10.3389/fbioe.2022.1023227

**Published:** 2022-10-05

**Authors:** J. F. Buyel, E. Benvenuto, A. E. Meyers

**Affiliations:** ^1^ Department of Biotechnology (DBT), Institute of Bioprocess Science and Engineering (IBSE), University of Natural Resources and Life Sciences, Vienna (BOKU), Vienna, Austria; ^2^ Institute for Molecular Biotechnology, RWTH Aachen University, Aachen, Germany; ^3^ Fraunhofer Institute for Molecular Biology and Applied Ecology IME, Aachen, Germany; ^4^ Italian National Agency for New Technologies, Energy and Sustainable Economic Development (ENEA), Technical Unit for Radiation Biology and Human Health (UTBIORAD), Rome, Italy; ^5^ Department of Molecular and Cell Biology (MCB), Biopharming Research Unit (BRU), University of Cape Town, Cape Town, South Africa

**Keywords:** genetic engineering, phytochemicals, plant-made pharmaceuticals (PMPs), transient expression, recombinant protein, virus-like particle (VLP)

Plants and plant cells can be used to produce numerous small molecules as well as recombinant technical and biopharmaceutical proteins. As such, they provide options for sustainable bioeconomy and manufacturing. However, in contrast to microorganisms and mammalian cell cultures that are firmly embedded in various industries, such as the pharma sector, research and development in plant molecular farming (PMF) is often driven by small- to medium-sized academic institutions and research groups scattered around the globe (Fischer and Buyel, 2020). A hub to trigger exchange between these groups, facilitate interactions with the industry, and ultimately translate PMF into an economically viable and sustainable biotechnological production alternative, is the International Society for Plant Molecular Farming (ISPMF). Specifically, the latest developments in the field are presented and discussed on a regular basis at the ISPMF’s biennial conference, which serves as a gauge of the latest developments and hot topics. We are, therefore, grateful to have been able to organize this research topic for the 4th biennial conference, which was also the first all-digital meeting due to pandemic restrictions. Because presenting the latest research results, even at an early stage, is encouraged at the conference, the articles included in this research topic reflect only a fraction of the data presented at the conference, which were mature enough to be published.

The research topics presented and discussed at the conference included the production of small molecules in plants, such as pheromones, triterpenic acids, and cyanophycin (Huckauf et al.). The production of recombinant proteins to be used in technical and food applications and for healthcare purposes such as therapeutic agents, e.g., antibodies and vaccines, was equally important. A special focus in this context was on the production of virus (-like) nanoparticles used to display epitopes (especially SARS-CoV-2) or antibody derivatives (Martí et al.) or used for technical applications such as metallization (Saunders et al.). An intensive discussion was carried out about the general expression system options such as cell-free systems, cultivating cells in bioreactors (Ruiz-Molina et al.), and the use of hairy roots. Along that line, the latest developments in terms of genetic engineering, e.g., using CRISPR/Cas, and the potential applications such as modified glycosylation and the corresponding outcomes were highlighted.

We expect that the findings presented at the conference and included in this research topic will make a substantial contribution to the advancement of the field.

## References

[B1] Fischer R. Buyel J. (2020). Molecular farming – the slope of enlightenment. Biotechnol. Adv. 40, 107519. 10.1016/j.biotechadv.2020.107519 31954848

